# Analysis of Fatty Acids, Amino Acids and Volatile Profile of Apple By-Products by Gas Chromatography-Mass Spectrometry

**DOI:** 10.3390/molecules27061987

**Published:** 2022-03-19

**Authors:** Anca Corina Fărcaș, Sonia Ancuța Socaci, Maria Simona Chiș, Francisc Vasile Dulf, Paula Podea, Maria Tofană

**Affiliations:** 1 Department of Food Science, Faculty of Food Science and Technology, University of Agricultural Sciences and Veterinary Medicine of Cluj-Napoca, 3–5 Mănăștur Street, 400372 Cluj-Napoca, Romania; anca.farcas@usamvcluj.ro (A.C.F.); maria.tofana@usamvcluj.ro (M.T.); 2 Department of Food Engineering, Faculty of Food Science and Technology, University of Agricultural Sciences and Veterinary Medicine of Cluj-Napoca, 3–5 Mănăștur Street, 400372 Cluj-Napoca, Romania; 3 Department of Environmental and Plant Protection, Faculty of Agriculture, University of Agricultural Sciences and Veterinary Medicine of Cluj-Napoca, 3–5 Mănăştur Street, 400372 Cluj-Napoca, Romania; francisc.dulf@usamvcluj.ro; 4 Department of Chemistry, Faculty of Chemistry and Chemical Engineering, Babeș-Bolyai University, Kogălniceanu, 400084 Cluj-Napoca, Romania; mpaula@chem.ubbcluj.ro

**Keywords:** apple by-products, fatty acids, amino acids, gas chromatography, volatile profile

## Abstract

Apple industrial by-products are a promising source of bioactive compounds with direct implications on human health. The main goal of the present work was to characterize the Jonathan and Golden Delicious by-products from their fatty acid, amino acid, and volatile aroma compounds’ point of view. GC-MS (gas chromatography-mass spectrometry) and ITEX/GC-MS methods were used for the by-products characterization. Linoleic and oleic were the main fatty acids identified in all samples, while palmitic and stearic acid were the representant of saturated ones. With respect to amino acids, from the essential group, isoleucine was the majority compound identified in JS (Jonathan skin) and GS (Golden skin) samples, lysine was the representant of JP (Jonathan pomace), and valine was mainly identified in GP (Golden pomace). A total number of 47 aroma volatile compounds were quantified in all samples, from which the esters groups ranged from 41.55–53.29%, aldehydes 29.75–43.99%, alcohols from 4.15 to 6.37%, ketones 4.14–5.72%, and the terpenes and terpenoids group reached values between 2.27% and 4.61%. Moreover, the by-products were valorized in biscuits manufacturing, highlighting their importance in enhancing the volatile aroma compounds, color, and sensorial analysis of the final baked goods.

## 1. Introduction

Apples (*Malus domestica* Borkh.), a member of the *Rosaceae* family, represent one of the most consumed fruits with a worldwide production of 86.1 million tons per year in 2018 [1] occupying third place after bananas and watermelon production [2,3].

They are consumed as fresh fruits or can be used for apple juice, jam, cider, and vinegar manufacture generating high amounts of residue, entitled apple pomace [4]. Apples are considered low-calorie fruits, extremely rich in vitamins, dietary fiber, minerals, phenols, and acids, and are able to prevent some diseases such as cancer, cardiovascular disease, or asthma [3]. Moreover, apple phenolic compounds and triterpene acids exhibit anti-inflammatory properties and have shown protective effects against Alzheimer’s disease [5].

Apple pomace (AP) represents 25% of the fresh apple weight and is an important by-product rich in dietary fiber, pectin, polyphenols, and minerals [1]. Only the apple juice industry claims to generate an annual AP quantity of about 10 million tones [6]. It seems that every liter of conventional juice processing generates over 300 g of AP [7].

AP is mainly composed of apple pulp, seeds, skin, and stalks, with a large moisture content (80% fresh weight) [8]. Due to its high moisture and sugar content such as sucrose, fructose, glucose, and xylose, the AP storage is difficult to realize. The most effective method of storing AP is through drying at mild temperature, without significantly affecting its bioactive compounds [7]. This by-product could be valorized as a gelling ingredient through the extraction of pectin or as a natural color pigment in the food industry, while polysaccharides such as cellulose and hemicellulose can be used in the paper-making industry or as a food additive, respectively [8,9]. The use of AP in chocolate manufacturing, aiming to partially replace sucrose, was recently studied by Büker et al. [10], while Masoodi et al. [11] and Sudha et al. [12] valorized AP in the manufacture of cakes as a source of dietary fiber and polyphenols, respectively. Furthermore, AP consumption can improve human gastrointestinal health decreasing the excretion of lithocholic acid and can have positive effects on cholesterol levels and inulin sensitivity [13].

Moreover, recently, AP was successfully used in the production of propionic acid, bioethanol, biogas, and value-added products such as aroma compounds, enzymes, or single-cell protein, but, unfortunately, even today, most of the AP amount is considered waste and disposed of in landfills [7,14,15].

Golden Delicious apples are preferred by consumers mainly because of their sweetness, color, firmness, intensive flavor, and light crunchiness [16]. They were characterized by Acquavia et al. [17] as having a golden yellow color, a crunchy and juicy pulp with a very sweet flavor being mainly used for juice, cider, jams, and canned goods manufacturing, having, as a substantial disadvantage, compared with other varieties, thin skin. Due to this drawback, they have a significant tendency to dehydration and more attention should be paid to their storage [18]. On the other hand, Jonathan apples are mainly used in fresh and frozen apple pies manufacturing, salads, apple sauce, and cobblers due to their specifical texture and moderate tart characteristics [19].

A substantial amount of literature describes the AP bioactive molecules such as polysaccharides, polyphenols, vitamins, dietary fiber, and minerals [3,4,5,10,14,15,20,21,22,23,24,25,26,27,28,29], but as far as we know, there is a lack of knowledge regarding its content in fatty acids, amino acids, and especially, aroma volatile compounds. Analytical techniques such as HPLC (high-performance liquid chromatography), HS-SPME (headspace solid-phase microextraction) coupled with GC-MS (gas chromatography-mass spectrometry), GC-MS, and HS–SPME/GC–qMS (headspace–solid-phase microextraction gas chromatography combined with quadrupole mass spectrometry) were used by a large body of literature for the identification and quantification of amino acids and aroma volatile compounds, respectively [30,31,32,33]. Fatty acids were identified and quantified through GC-MS or GC-FID (gas chromatography coupled with flame ionization detector) according to [16,34,35].

**Figure 1:**
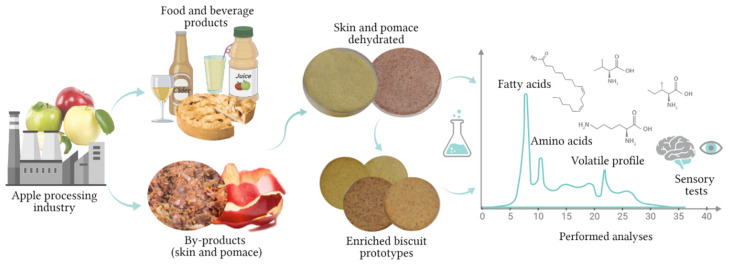
Experimental design of the present work.

Therefore, the aim of the present study was to characterize Golden Delicious and Jonathan apple by-products such as apple pomace and skin from their amino acids, fatty acids, and aroma volatile compounds’ point of view. Furthermore, the addition of 25% AP and skin in biscuits and its influence on their sensorial analysis, color, and aroma volatile compounds were also studied, giving new insights into AP and skin valorization in the food industry. The experimental design of the present study is briefly illustrated in Figure 1.

## 2. Results

### 2.1. By-Products Fatty Acids, Amino Acids and Volatile Profiles

#### 2.1.1. By-Products Fatty Acids Content

**Figure 2:**
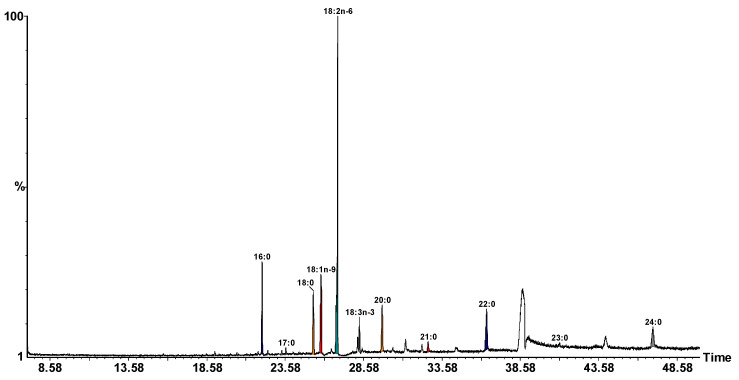
GC-MS chromatogram of FAMEs in the TLs of dried JP analyzed on a SUPELCOWAX 10 capillary column. Peaks: Palmitic, (16:0); Z-7-Hexadecenoic, 16:1(*n*−9); Margaric,17:0; Stearic, 18:0; Oleic, 18:1(*n*−9); Vaccenic, 18:1(*n*−7); Linoleic, 18:2(*n*−6); α-Linolenic, 18:3(*n*−3); Arachidic, 20:0; Heneicosanoic, 21:0; Behenic, 22:0; Tricosanoic, 23:0; Lignoceric, 24:0.

Linoleic acid, the main representant of the PUFA (polysaturated fatty acids) group, was identified in considerable amounts in JS (Jonathan skin) and GS (Golden skin) samples with values of 85.08% and 83.44%, whilst the JP (Jonathan pomace) and GP (Golden pomace) samples registered values of 37.86% and 38.27%, respectively. From the SFA (saturated fatty acids) group, palmitic acid was mainly identified in the JS and GS samples (Table 1). With respect to GS, linoleic acid was the main fatty acid, but with a significantly smaller amount (83.44%) compared with JS (85.08%), followed by oleic (7.61%) and palmitic (2.97%) fatty acids. Regarding MUFA (monosaturated fatty acids), the main amount was identified in GP and JP, followed by GS and JS, as displayed in Table 1. Figure 2 displays the JP chromatogram.

#### 2.1.2. By-Products Amino Acids Content

The amino acid samples content is displayed in Table 2. For a better explanation, the 14 identified amino acids were divided into two groups: essential (EAA) and non-essential (NEAA) ones. The identified essential amino acids are: threonine (Thr), valine (Val), leucine (Leu), isoleucine (Ile), methionine (Met), phenylalanine (Phe), lysine (Lys), while alanine (Ala), glycine (Gly), serine (Ser), γ-aminobutyric acid (GABA), proline (Pro), asparagine (Asp), and glutamic acid (Glu) are considered NEAA amino acids. On the other hand, according to Chiș et al. [36] and Katina et al. [37], amino acids can be divided into five groups: aliphatic, which include prolamine, valine, leucine, glycine, alanine, aromatic (phenylalanine), acids (glutamic acid and aspartic acid), γ-aminobutyric acids (serine, threonine, proline, methionine, and γ-aminobutyric) and basic group (lysine).

With respect to the total amino acids content, GP was the richest sample with a value of 94.38 mg/100 g, followed by JP with a value of 87.37% and 60.58% and 56.82% for GS and JS, respectively. 

From the essential group, the highest value was identified in JS (12.25 mg/100 g), followed by GS (10.86 mg/100 g), while JP and GP reached values of 9.95 mg/100 g and 7.37 mg/100 g, respectively. The main JS representants of EAA were Ile (3.41 mg/100 g), followed by Val (3.14 mg/100 g) and Lys (1.82 mg/100 g), while higher amounts were registered by NEAA with Asp (28.10 mg/100 g), Gly (6.66 mg/100 g), and Ala (4.09 mg/100 g). Asp (31.05 mg/100 g), Gly (7.57 mg/100 g), and Ala (5.14 mg/100 g) were the majority representants of NEAA from GS, while Ile (4.14 mg/100 g), Lys (1.56 mg/100 g), and Met (1.32 mg/100 g) were principal compounds of the EAA group. Lysine, one of the first aliphatic-limiting cereals amino acids [38], was mainly identified in JP and JS was statistically different (*p* < 0.05) from GP and GS, respectively.

#### 2.1.3. Apple By-Products Aroma Volatile Profile

The aroma volatile compounds are displayed in Table 3. A total number of 48 compounds have been identified and divided into the groups: alcohols, esters, aldehydes, ketones, terpenes, acids, and others. The most representative group was that of esters, ranging between 44.81% to 53.29%, followed by aldehydes (29.75% to 43.99%) and ketones (4.14% to 5.72%). The main compound from the aldehydes group was hexanal, scoring the highest value for GP (20.68%), while from the esters group, butyl acetate reached the highest value of 19.47% for the JS sample. With respect to the alcohols group, the most important compound was 1-hexanol, ranging from 2.18% to 4.11%, while acetophenone and 6-methyl-5-hepten-2-one were the main representants from the ketones group. From terpenes, α-farnesene and D-limonene, responsible for wood, sweet, floral, and citrus, fresh odors perception, were mainly identified in the JS sample with values of 1.54% and 1.5%, respectively.

### 2.2. Biscuit’s Aroma Profile, Color Characteristics and Sensory Analysis

#### 2.2.1. Biscuit’s Aroma Profile

A total number of 20 aroma volatile compounds were identified in the final baked goods, as follows: 2 alcohols, 4 esters, 8 aldehydes, 2 ketones, 2 terpenes, and terpenoids, 1 acid, and 1 other compound (Ethyl 2,4-dioxohexanoate), as presented in Table S1, Supplementary Materials. From the esters group, acetic acid (14.96%) was only identified in BCS, while in the BJS sample, hexyl acetate scored the highest value of 14.8%. From the aldehydes group, hexanal was mainly present in BCS, while benzaldehyde was the main representant of the BJS sample; 2-heptanone was the major compound of the ketones group with the highest score in the BJS sample (13.99%) and D-limonene (2.99%) was the main representant of the terpenes and terpenoids group.

#### 2.2.2. Biscuits and By-Products Color Characteristics

**Figure 3:**
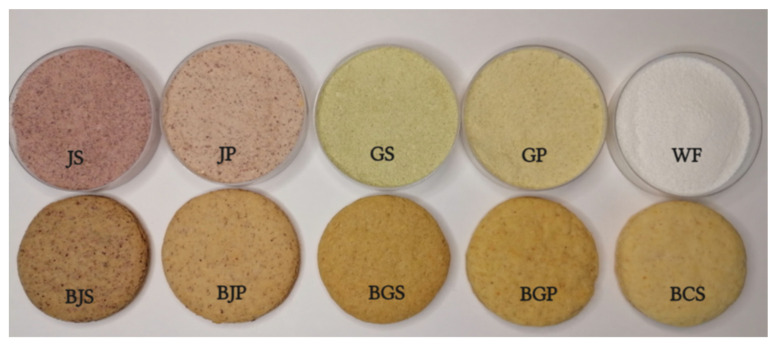
By-products and final baked biscuits. BJS: biscuits with JS; JS: Jonathan skin; BJP: biscuits with JP; JP: Jonathan pomace; BGS: biscuits with GS; GS: Golden skin; BGP: biscuits with GP; BCS: biscuits with WF; GP: Golden pomace; BCS: biscuits control sample; WF: wheat flour.

The color parameters of the final baked products and by-products are displayed in Table 4 and illustrated in Figure 3, showing the final aspect of the by-products and final baked goods.

The highest L* value was reached by the biscuits control sample (BCS), while the highest a* value was registered by biscuits manufactured with BJS. With respect to the b* parameter, significant differences were registered between all samples (Table 4). With respect to apple by-products, parameter a* had the highest value in the JS sample, while the lowest value was registered for the GS sample. Contrariwise, the b* parameter registered values ranging from 15.22 to 29.50, the biggest one being highlighted by the GS sample. With respect to the L* parameter, there were significant differences between the sample (*p* < 0.05), as presented in Table 4.

#### 2.2.3. Sensory Analysis

**Figure 4:**
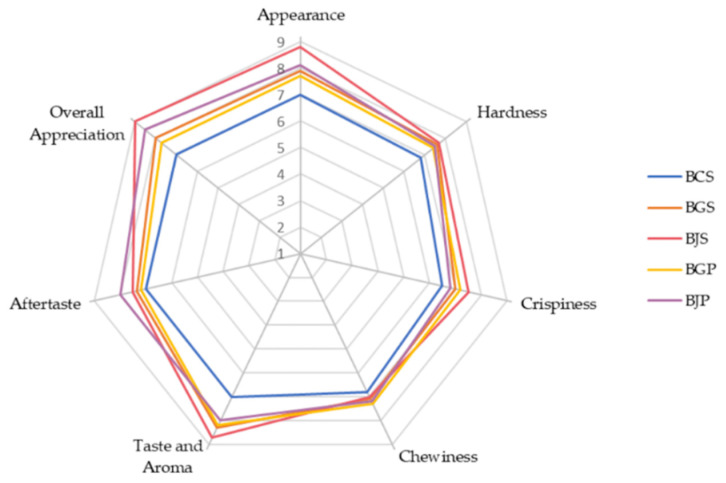
BCS—biscuits control sample; BJS—biscuits with JS; BJP—biscuits with JP; BGS—biscuits with GS; BGP—biscuits with GP.

The final baked goods were evaluated by panelists with respect to their appearance, taste and aroma, hardness, crispiness, chewiness, aftertaste, and overall appreciation. The highest score for taste and aroma, appearance, overall appreciation, and aftertaste were registered by the BJS sample, while the control sample registered the lowest value, as presented in Figure 4. Textural parameters such as hardness, crispiness, and chewiness decreased through AP addition, being significantly different compared to the control sample. Hardness is characterized as the force of the first compression cycle, while crispiness is defined as the combination between force and noise caused by the molar teeth when breaking down the sample, [39]. Chewiness is defined as the difficulty level needed for a panelist in order to chew the sample and to form the bolus before the swallowing process [40].

## 3. Discussion

The cultivation conditions and techniques, species, and variety have a significant impact on the chemical composition of apples [3]. The quality of apples is related also to their fatty acids and free amino acids content [41]. Fatty acids are crucial components of the fruit cell membrane being involved in the majority of physical, functional, and chemical reactions and their imbalance could lead to different storage fruits disorders [42]. 

In the present study, C18 family fatty acids accounted for more than 91.59%, 91.42%, 65.79, and 65.10% for GS, JS, JP, and GP, respectively. Results are consistent with Wu et al. [35] who showed that the C18 family registered more than 70% of the total fatty acid content in eight apple cultivars from Shandong Province, China. Recently, Di Matteo et al. [2] showed that in apples from the Piedmont region, Italy, polyunsaturated fatty acids were the most abundant ones ranging between 30% and 45%, and the monounsaturated fatty acids content was between 5% and 25%. Moreover, authors reported significant differences between fatty acids samples content, showing that, for instance, Canditina cultivar had the highest concentration of unsaturated fatty acids, while Dominici registered the lowest value.

Linoleic, α-linolenic, and oleic acids were also identified in apple pomace by several authors such as Radenkovs et al. and Rodríguez et al. [42,43], while Dadwal et al. [31] showed that the wild crab seed apples (*Malus baccata*) from the Himalayan region were mainly rich in palmitic acid, ethyl palmitate, and linolein. In line with this, Rodríguez et al. [43] showed that the main identified fatty acids in apple pomace were linoleic and oleic acids, which represent more than 70% of the total fatty acids amount.

Recently, Lamani et al. [34] showed that wood apples (*Limonia acidissima* L.) collected from India contained 51.98 ± 0.94% unsaturated fatty acids from which the most abundant were oleic acid (23.89 ± 0.06%), α-linolenic, and linoleic acid with percentages of 16.55 ± 0.26% and 10.02 ± 0.43%, respectively.

According to Berto et al. [44], a PUFA/SFA ratio larger than 0.45 value is considered positive for human health, while a value smaller than 0.45 could lead to an increased blood cholesterol level. In the present study, all samples registered values larger than 0.45 ranging from 0.94–9.82%.

Considering the above, we can assess that the extraction procedure, method used for analysis, pedo-climatic conditions, genetic factors, and apple maturity stage are the main factors that could influence the fatty acid by-product total amount. For instance, fatty acids could be extracted with different solvents such as hexane or petroleum ether and be further analyzed through GC-FID (gas chromatography coupled with flame ionization detector) or GC-MS (gas chromatography coupled with mass spectrometry) [17].

Amino acids are defined as essential biomolecules with a tremendous role in tissue protein blocks and human health. They are claimed to have positive results in diseases such as infertility, intestinal disorders, and neurological dysfunction and could be used as fingerprints to uncover the fruits varietal origin [45,46].

Free amino acids are defined as fatty acids that result from lipase activity that are metabolized by enzymes such as β-oxidative and lipoxygenase, which are the main precursors of aroma compounds (e.g., esters, alcohols, and aldehydes). The harvesting time is a crucial factor involved in the aroma of apple production, with earlier harvesting causing a decrease in the aroma spectrum [42]. Amino acids are considered aroma precursors during fruit maturation and are utilized for the synthesis of aroma components [47], being the second most important source in the development of volatile aroma compounds [48]. Free amino acids are important for food flavoring, improving its palatability, and helping in the development of amines and volatile compounds [49]. For instance, tyrosine and phenylalanine can be substrates for the further development of aroma compounds [41]. The presence of amino acids such as Gly, Ala, and Pro influences the taste of fruits in a positive way, providing sweetness [47].

In the present study, Asp was the most abundant amino acid identified in all samples, ranging from 28.10 to 63.57 mg/100 g. Recently, it has been shown that Asp could be successfully used in the prevention of diabetic kidney mice disease, highlighting the importance of this non-essential amino acid in kidney oxidative stress reduction [50]. Asp was identified in high amounts also by Zhang et al. [51] at the maturity stage of Honeycrisp apples, but also as the second most abundant amino acid in apple *Malus domestica* Borkh cv. Annurca, a variety from Southern Italy [52].

On the other hand, in our study, Gly was the second most identified amino acid from 6.66 to 14.24 mg/100 g. It was mentioned by Mosa et al. [53] that using Gly and tryptophan as alternatives for chemical fertilizers leads to an improvement in apple quality playing a key role in increasing the total chlorophyll amount and viability of some minerals. Alanine was the third most abundant amino acid in the analyzed samples and was also identified by Di Matteo et al. [2] in apples from the Piedmont region, Italy, ranging from 1 to 11 mg/100 g for *Canditina* and *Grenoble* cultivars, respectively. On the other hand, Dadwal et al. [31] mentioned that in the pulp extract of *Malus baccata* crab apple, amino acids such as tyrosine, cysteine, glycine, alanine, serine, and histidine were identified.

It is worth noting that recently, more attention is being paid to γ-aminobutyric acid which is formed via the enzymatic reaction of glutamic acid with several important roles in human metabolism such as hypotensive, diuretic, neurotransmitter, and inhibitor of leukemia cell proliferation [54,55]. Furthermore, in all samples, Val, Ile, and Leu were identified and claimed by the literature to have an essential role in human muscle damage recovery, fatigue, and soreness due to physical effort. Val, Ile, and Leu are entitled branched-chain amino acids and are essential amino acids that are able to stimulate insulin production, prevent or even cure hepatic encephalopathy, and act as neurotransmission modulators [56].

Wicklund et al. [33] mentioned that the accumulation of amino acids such as aspartic and glutamic acid are in direct correlation with horticultural conditions, mainly the presence of nitrogen. More broadly, cultivar and year of cropping could influence the apple fruit composition, considering their primary and secondary metabolites [57]. Strengthening this idea, di Marro et al. and Eleutério et al. [30,52] underlined that water stress, mineral nutrition, fruit maturity, pedo-climatic conditions such as light, and soil treatments (fertilization with nitrogen) could affect the amino acid amount. Di Maro et al. [52] identified a total amount of amino acids from 10 apple cultivars ranging from approximately 1 mg/100 g of dry weight to 340 mg/100 g dry weight, emphasizing once again the difference of total amino acids due to their varieties.

Apple chemical compounds and the ratio between them are considered the main factors that could influence the flavor, taste, consistency, and health benefits [2]. For instance, taste is mainly influenced by sugars and the organic acid content, and aroma by the volatile profile [48]. On the other hand, fatty acids and lipids play a key role as precursors of aroma volatile compounds [23]. Oleic and linoleic acids emphasized a strong relationship with aroma production [41]. It was stated that the unsaturated fatty acids are directly correlated to the storage and release of aroma components, acting as flavor precursors [34].

In the present study, esters were the main aroma compounds identified in JS, GP, GS, and aldehydes in the JP sample. The presence of esters in apples such as butyl acetate, 2-methylbutyl acetate, hexyl acetate, and 2-methylbutanoate was also claimed by Espino-Díaz et al. [48] as the principal esters with a high impact on the final apple aroma. In line with this, Coelho et al. [32] mentioned that the major volatile aroma compounds of industrial apple aroma were composed of esters and aldehydes. Moreover, from our results, a strong Pearson correlation was identified between esters and fatty acids and fatty acids and aldehydes, respectively. For instance, in the JS and GS samples, correlations of 0.998 and 0.997 were identified between linoleic acid and esters and aldehydes, respectively. The same trend was observed between JP and GP, highlighting once again the strong relationship between linoleic acid, esters, and aldehydes. The results are explained by the fact that unsaturated fatty acids play a paramount role in aroma apple development, as described by a large body of literature [34,41,48].

Aldehydes are formed as a result of two different reactions: fatty acid catabolism and the metabolism of branched-chain amino acids such as valine, leucine, isoleucine. Alcohols are released through the reduction of aldehydes by the alcohol dehydrogenase enzyme [48]. Aldehydes are correlated with the ripening stage of apples, and decrease during the maturity stage, leading to the formation of esters and alcohols [48]. 

The presence of alcohols is generally explained as a result of the fermentation between amino acids and carbohydrates, but the presence of 1-hexanol, which is responsible for green, sweet, herbaceous, fermented notes, fruity, apple-skin, and oily, is varietal [58]. On the other hand, 1-butanol, which possesses sweet, balsamic, oily, and whiskey aromas, is involved in a positive way in the aroma characteristics and intensity of apples [59].

From the terpenes and terpenoids group, a total of 12 volatile compounds were identified, from which D-limonene, 1,3,8-p-menthatriene, α-farnesene, and camphene were identified in all samples. Moreover, as far as we know, for the first time in the literature, compounds such as terpinolene, 3-carene, sabinene, ß-pinene, ß-myrcene, and 1,3,8-p-menthatriene were identified in AP by-products.

With respect to the volatile biscuit’s aroma compounds, BCS (biscuits control sample) registered the lowest amounts for esters, ketones, and terpenes and terpenoids, mainly because wheat flour is not a rich raw material in aroma volatile compounds. In line with this, our recent publication Fărcaș et al. [60] identified only five aroma volatile compounds from wheat flour, mainly composed of aldehydes and ketones in percentages of 91.99% and 8.02%, respectively. The presence of aldehydes and ketones could be explained by the non-enzymatic Maillard reaction, as a consequence between amino acids and sugars [60].

The presence of 3-metyl-butanal, 2-methyl-butanal, and 2-methyl-propanal in the samples manufactured with apple by-products could be attributed to the branched-chain amino acids such as leucine, isoleucine, and valine, which are claimed to be involved in the aforementioned aroma volatile compounds synthesis [61,62]. Strong Pearson’s correlations were identified between the total amount of branched-chain amino acids and the total amount of the three mentioned aldehydes. For instance, between the JS and JP total amount of branched-chain amino acids and BJS and BJP 3-metyl-butanal, 2-methyl-butanal, and 2-methyl-propanal total amount, a strong relationship of 0.998 was identified. A strong Pearson correlation (0.997) was identified also in GS and GP samples between the aforementioned amino acids and BGS and BGP aldehydes, respectively.

Moreover, recently, Garvey et al. [62] suggested that the presence of aldehydes such as phenylacetaldehyde and methional in the final baked samples could be explained through the presence of amino acids such as phenylalanine and methionine in apple by-products (Table 2). The phenylacetaldehyde compound is responsible for sweet, rose, or honey aroma, while methional is characterized as being responsible for exhibiting a potato-like odor [62]. From the ketones group, 2-heptanone scored the highest value from the BJS and BGP samples, being responsible for cheese, fruity, ketonic, green banana, with a creamy nuance odor. It is worth noting that D-limonene was identified mainly in the BJS and BJP samples, providing the final baked samples odor perceptions such as citrus, fresh, and sweet. It is also important to mention that apple by-products are a rich source of sugars such as fructose, glucose, sorbitol, and saccharose [15], enhancing the Maillard reaction and therefore, facilitating the development of new aroma volatile compounds.

The Jonathan red coloration skin is mainly due to the presence of anthocyanins, a class of flavonoids that are directly influenced by genetic factors and pedo-climatic conditions such as temperature, light, and nutrition [63]. According to Honda et al. [63], there are five genes responsible for the red coloration in apples and at the ripe final stage, the genes reached the highest expression levels. In line with this, Melnic et al. [64] supported the idea that anthocyanin compounds are responsible mainly for the peel redness apple color and further studies are still needed to better elucidate the mechanism. With respect to Golden Delicious apple color, higher values of b* are related to higher amounts of carotenoids and xanthophylls which are a result of the decrement in greenness appearance and increasement in yellowness through the apple ripening stage [65]. 

The WF (wheat flour) substitution with apple by-products caused a change in color on the final baked products—biscuits becoming darker and redder. The redness value (a*) of the BJS sample reached the highest value, being significantly different from the other samples; while the lowest value was represented by the control sample (BCS). This could be explained by the chemical composition of JS, rich in anthocyanins which are responsible for the red color. In red apples, cyanidin is the most representative anthocyanin [66]. With respect to yellowness (b* value), the highest value was registered for the BGS sample and could be due to the Golden delicious skin color. The color of Golden Delicious apples is explained by their rich flavanols content, claimed to be responsible for the yellow color of apple skins [67]. Furthermore, Golden Delicious is described as an apple with low browning potential [68]; therefore, the color of the final baked goods is lighter than those manufactured with the Jonathan variety. The results are in line with those of Sudha et al. [69] and Jung et al. [7] who showed that the AP addition in bakery products caused changes in the color of the final baked goods.

The appearance of the final baked goods was significantly different (*p* < 0.05), and the BJS sample recorded the highest score (Figure 4). It seems that panelists gave the highest score to the darker samples, probably considering it healthier. The idea that products with a darker color are healthier than conventional ones is supported by the literature [60,70,71]. For instance, Drogoudi et al. [70] showed through PCA (principal component analysis) and correlation analysis between seven apple varieties and their phenolic and antioxidant activity that apple skin with a darker, redder, or bluer color are more nutritious than the uncolored ones.

With respect to taste and aroma, all final baked samples were accepted by consumers reaching scores ranging from 8.2−8.7, while the control samples registered a value of only 7. This could be justified by the AP aroma. According to Sudha et al. [12], cakes manufactured with increased levels of AP were considered by panelists as having a pleasant fruit flavor.

The hardness value of biscuits was significantly different from the control sample (*p* < 0.05). This could be justified by the apple fiber content and by gluten reduction through replacing WH with AP. With respect to the crispiness value, a significantly increased value was observed in AP biscuits compared with the control sample, while chewiness slightly increased compared to the control sample. This could be explained by the AP-rich fiber content (with a value in the range of 4.4–47.3% fresh weight) which, according to Skinner et al. [13], has strong water-binding properties [12]. Furthermore, Sudha et al. [12] mentioned that AP fibers are considered to be superior to oat bran and wheat, having a better quality of dietary fiber.

## 4. Materials and Methods

### 4.1. Materials, Reagents

Golden Delicious and Jonathan apples were purchased from a local supermarket in Cluj-Napoca, having Romania as the producer country. The Jonathan and Golden apple skins were provided from the bakery and pastry pilot station from the University of Agricultural Sciences and Veterinary Medicine, Faculty of Food Science and Technology from Cluj-Napoca, Romania. In this pilot station, there is a daily production of a total amount of 40 kg apple cakes, resulting in approximately 10 kg of apple skins that are discarded and considered waste (according to the apple cake recipe, data not shown). On the other hand, a beer pilot station from the same faculty produces an annual amount of 250 L apple cider, manufactured with Golden and Jonathan apples. The residue obtained after the apples were pressed to obtain the juice is generally entitled apple pomace (mainly composed of apple pulp and skin) and is also discarded as food waste. All reagents were analytical grade and purchased from Sigma-Aldrich (Steinheim, Germany), as presented in Table S2.

### 4.2. Apple Pomace (AP) and Biscuits Manufacturing

**Figure 5:**
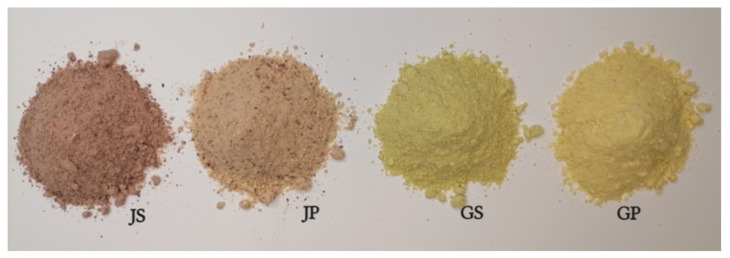
Apple by-products powders; JS: Jonathan skin; JP: Jonathan pomace; GS: Golden Delicious skin; GP: Golden Delicious pomace.

Apple pomace (AP) and apple skin were dried at a temperature of 55 °C using a professional dehydrator (Hendi Profi Line, Utrecht, The Netherlands) and ground through a laboratory professional mill (IKA A10, Staufen, Germany). Afterward, the AP and skins were sieved through a sieve (0.42–0.60 mm) in order to obtain a fine powder, as illustrated in Figure 5.

The biscuits manufacturing was carried out according to our previous research studies [39,60]. The vegetable fat was first mixed with sugar using an automatic mixer (Kitchen Aid Precise Heat Mixing Bowl, Greenville, OH, USA), low speed, until a cream base was obtained. The wheat flour (WF) was manually mixed with baking powder and AP and skin, respectively. The WF that replaced 25% of AP and skin was based on our previously obtained products and considered from already publicized articles [69,72]. The technological parameters and biscuits recipes are presented in Table 5. A thickness dough of 0.8 cm was obtained by using a semiautomatic laminator (Flamic SF600, Vicenza, Italy). After baking in an electric oven (Zanolli, Verona, Italy), the samples were cooled down at bakery pilot station temperature and used for further analysis. From a microbiological point of view, the safety of the final baked goods was according to Romanian Regulations (Order No. 27/2011) and SR ISO 21527-2/2008 standard, [73,74].

### 4.3. Fatty Acids

Folch’s total lipids extraction procedure was carried out according to the method described by [75] and [76]. Briefly, 3 g of samples was mixed for 1 min with 5 mL of methanol using a high-power homogenizer (MICCRA D-9, ART Prozess-und Labortechnik, Mullheim, Germany). Afterward, 10 mL of chloroform was added, and the homogenization process continued for 2 more minutes. A solution with chloroform/methanol (2:1, *v*/*v*, 15 mL) was used for the re-extraction of the solid residue, previously filtered. The resulted filtrates were washed with 0.88% aqueous potassium chloride in a separation funnel to purify the lipids, dried over anhydrous sodium sulphate, and the solvent was removed through a rotary evaporator (Rotavapor R-124, Buchi, Flawil, Switzerland).

Total lipids fatty acid methyl esters (FAMEs) were analyzed as described by Fărcaș et al. [77] through GC-MS (gas chromatography-mass spectrometry) using a PerkinElmer Clarus 600 T GC-MS (PerkinElmer, Inc., Shelton, CT, USA) equipped with a Supelcowax 10 capillary column (60 m × 0.25 mm i.d., 0.25 µm film thickness; Supelco Inc., Bellefonte, PA, USA). The initial temperature of the column was 140 °C and reached a final temperature of 220 °C through an increase of 7 °C/min. The final temperature was kept for 23 min. Helium was used as the carrier gas with a flow rate of 0.8 mL/min and mass spectra were recorded in EI (positive ion-electron impact) mode with mass scans performed in the range of 22 to 395 *m*/*z*. FAMEs were identified by comparing their retention time with those of the known standards (37 components FAME Mix, Supelco No. 47885-U) and the obtained mass spectra with those from the NIST MS Search 2.0 software database (Gaithersburg, MD, USA). The amount of each fatty acid was expressed as the peak area percentage of total fatty acids.

### 4.4. Amino Acids

A DSQ Thermo Finnigan quadrupole mass spectrometer coupled with a Trace GC was used for the analytical investigation. The samples were dried and crushed and 100 mg of each sample was extracted with 1 mL of 6% trichloroacetic acid in an ultrasonic bath and then purified on an ion-exchange solid phase column, as described by Culea et al. [51]. Quantitation of amino acids was performed by adding 15N-glycine 99 atom % as an internal standard. Amino acids were derivatized as trifluoroacetic butyl esters, separated on a nonpolar capillary chromatographic column (Rtx-5MS capillary column: 30 m × 0.25 mm, 0.25 mm film thickness) with the following temperature program: 70 °C, 2 min, 5 °C/min to 110 °C, 10 °C/min to 290 °C, and 16 °C/min to 300 °C. Helium was used as the carrier gas with a flow rate of 1mL/min, under the next conditions: ion source temperature of 250 °C, injector temperature 200 °C, splitter: 10:1, electron energy of 70 eV and with a line transfer temperature of 250 °C.

### 4.5. Aroma Volatile Compounds

The extraction of aroma volatile compounds was performed through the in-tube extraction technique (ITEX) and the analysis was carried out on a GCMS QP-2010 gas chromatograph-mass spectrometer instrument (Shimadzu Scientific Instruments, Kyoto, Japan) as described in our previous works [77,78]. Briefly, 3 g of each sample was introduced into a headspace vial of 20 mL, incubated for 20 min at a temperature of 60 °C, and the volatile compounds in the gas phase were absorbed through a fiber syringe (ITEX-2TRAPTXTA, Tenax TA 80/100 mesh) and directly desorbed into the GC-MS injector.

A Zebron ZB-5MS (Phenomenex) capillary column was used for the separation of the volatile compounds with helium as the carrier gas, a split ratio of 1:5, and a flow rate of 1 mL/min. The chromatographic column program was as follows: 35 °C (for 5 min) rising to 155 °C with 7 °C/min and then heated to 260 °C with 10 °C/min and held for 5 min. NIST27 and NIST147 mass spectra libraries were used for identifying the spectra of the reference compounds and checked by comparison with retention indices drawn from www.pherobase.com or www.flavornet.org [79,80]. The peaks that were identified at least in two of the three total ion chromatograms (TIC) were considered in calculating the total area of peaks (100%) and the relative areas of the volatile compounds [44].

### 4.6. By-Products and Biscuits Color Characteristics

The color characteristics of the final biscuits were analyzed according to our recent work [50], by using an NH 300 portable colorimeter (Shenzhen ThreeNH Technology Co., Ltd., Shenzhen, China), having a color system based on CIE L* (luminosity), a* (red/green coordinate), b* (yellow/blue coordinate) color. The colorimeter was previously calibrated using its own black and white calibration system. All measurements were made in triplicate and presented as the mean ± sd (standard deviation).

### 4.7. Sensory Analysis

Sensory analysis was carried out according to the method described in our previous study [60] and was based on a nine-point hedonic scale. The panelists were students or staff members of the Faculty of Food Science and Technology, and the sensorial analysis took place in a laboratory near the bakery pilot station. A total of 35 panelists participated in the analysis, previously selected due to their regular biscuit consumption, from which 25 were females and 10 were males. A nine-point hedonic scale was based on the following attributes of the final baked goods: appearance, hardness, crispiness, chewiness, and taste and aroma. The attributes were rated with notes from 1 to 9, in which 1 means extremely dislike and 9 was the maximum note meaning extremely like.

## 5. Conclusions

In the present study, a total number of 15 fatty acids, 14 amino acids, and 47 aroma volatile compounds were identified in apple by-products samples. Strong Pearson correlations were highlighted between branched amino-acids such as leucine, isoleucine, and valine and apple by-products volatile aroma compounds (mainly 3-methyl-butanal, 2-methyl-butanal, and 2-methyl-propanal). From the esters group, butyl acetate reached the highest value in JS (19.76%), while hexanal from the aldehydes group was the main representant of GS (20.68%). In the present research, terpinolene, 3-carene, sabinene, ß-pinene, ß-myrcene, and 1,3,8-*p*-menthatriene were first identified in apple by-products. The valorization of 25% apple by-products in biscuits manufacturing exhibited a positive influence on nutritional, volatile, and sensorial characteristics of the final baked goods.

## Figures and Tables

**Table 1:** Apple by-products fatty acids content.

Shorthand Nomenclature	Fatty Acid Name	Type	JS (%)	JP (%)	GS (%)	GP (%)
12:0	Lauric	SFA	0.08 ± 0.02 ^a^	n.d.	0.06± 0.01 ^a^	n.d.
14:0	Myristic	SFA	0.13±0.01 ^a^	n.d.	0.09 ± 0.02 ^a^	n.d.
16:0	Palmitic	SFA	3.83 ± 0.14 ^ab^	9.27 ± 0.31 ^c^	2.97 ± 0.06 ^a^	9.17 ± 0.15 ^c^
16:1 (*n*−9)	Z-7-Hexadecenoic	MUFA	n.d.	0.29 ± 0.02 ^a^	n.d.	0.27 ±0.02 ^a^
17:0	Margaric acid	SFA	0.39 ± 0.02 ^a^	0.70 ± 0.02 ^ab^	0.17 ± 0.02 ^a^	0.77 ± 0.03 ^ab^
18:0	Stearic acid	SFA	2.78 ± 0.21 ^a^	9.93 ± 0.34 ^b^	2.53 ± 0.57 ^a^	9.70 ± 0.05 ^b^
18:1 (*n*−9)	Oleic acid	MUFA	3.17 ± 0.03 ^a^	13.27 ± 0.05 ^c^	7.61 ± 0.25 ^b^	13.64 ± 0.05 ^c^
18:1 (*n*−7)	Vaccenic acid	MUFA	n.d.	0.11 ± 0.02 ^a^	n.d.	0.19 ± 0.02 ^a^
18:2 (*n*−6)	Linoleic acid	PUFA	85.08 ± 0.63 ^b^	37.86 ± 0.33 ^a^	83.44 ±0.31 ^c^	38.27± 0.03 ^a^
18:3 (*n*−3)	α-linolenic acid	PUFA	0.39 ± 0.02 ^a^	3.92 ± 0.05 ^b^	0.41 ±0.03 ^a^	3.99 ± 0.21 ^b^
20:0	Arachidic	SFA	1.36 ± 0.05 ^b^	7.52 ± 0.03 ^c^	0.58 ± 0.03 ^a^	7.26 ± 0.05 ^c^
21:0	Heneicosanoic	SFA	n.d.	1.88 ± 0.12 ^b^	0.08 ± 0.02 ^a^	1.93 ± 0.02 ^b^
22:0	Behenic acid	SFA	1.88 ± 0.03 ^ab^	9.32 ± 0.11 ^c^	1.34 ± 0.05 ^a^	9.46 ± 0.04 ^c^
23:0	Tricosanoic	SFA	n.d.	0.40 ± 0.03 ^a^	n.d.	0.43 ± 0.02 ^a^
24:0	Lignoceric	SFA	0.90 ± 0.03 ^a^	5.52 ± 0.05 ^bc^	0.72 ± 0.03 ^a^	4.92 ± 0.04 ^b^
∑ SFA			11.37 ± 0.69 ^b^	44.55 ± 0.99 ^c^	8.54 ± 0.74 ^a^	43.63 ± 0.40 ^c^
∑ MUFA			3.17 ± 0. 0.03 ^a^	13.67 ± 0.09 ^c^	7.61 ± 0.56 ^b^	14.11 ± 0.09 ^c^
∑ PUFA			85.47 ± 0.65 ^b^	41.78 ± 0.38 ^a^	83.85 ± 0.34 ^bc^	42.26 ±0.24 ^a^
∑ *n*−3 PUFA			0.39 ± 0.02 ^c^	3.92 ± 0.05 ^b^	0.41 ± 0.03 ^a^	3.99 ± 0.21 ^b^
∑ *n*−6 PUFA			85.08 ± 0.63 ^b^	37.86 ±0.33 ^a^	83.44 ± 0.31 ^bc^	38.27 ± 0.03 ^a^
∑ *n*−6/*n*−3			216.67 ^c^	9.66 ± 0.28 ^a^	203.30 ^b^	9.59 ^a^
∑ PUFAs/SFAs			7.52 ^b^	0.94 ^a^	9.82 ^c^	0.97 ^a^

JS: Jonathan skin; JP: Jonathan pomace; GS: Golden Delicious skin; GP: Golden Delicious pomace; different superscript letters in a row indicate significant difference between samples (*p* < 0.05).

**Table 2:** Apple by-products amino acid content.

Amino Acid Name	Type	JS mg/100 g	JP mg/100 g	GS mg/100 g	GP mg/100 g
Ala	NEAA	4.09 ± 0.03 ^a^	7.16 ± 0.05 ^d^	5.14 ± 0.04 ^b^	5.69 ± 0.07 ^bc^
Gly	NEAA	6.66 ±0.04 ^a^	14.24 ± 0.16 ^d^	7.57 ± 0.11 ^b^	12.53 ± 0.16 ^c^
Thr	EAA	0.57 ± 0.07 ^a^	0.56 ± 0.12 ^a^	0.88 ± 0.03 ^ab^	0.70 ± 0.03 ^a^
Ser	NEAA	0.78 ± 0.05 ^a^	1.24 ± 0.03 ^b^	1.17 ±0.09 ^b^	1.58 ± 0.05 ^bc^
Val	EAA	3.14 ± 0.08 ^d^	1.13 ± 0.05 ^a^	1.58 ± 0.05 ^ab^	2.21 ± 0.12 ^c^
Leu	EAA	1.14 ± 0.11 ^c^	1.71 ± 0.09 ^d^	0.72 ± 0.05 ^ab^	0.52 ± 0.03 ^a^
Ile	EAA	3.41 ± 0.06 ^c^	1.85 ± 0.08 ^b^	4.14 ± 0.11 ^d^	1.10 ± 0.06 ^a^
GABA	NEAA	1.06 ± 0.03 ^b^	0.40 ± 0.03 ^a^	3.38 ± 0.22 ^c^	1.01 ± 0.02 ^b^
Met	EAA	1.35 ± 0.02 ^a^	1.22 ± 0.11 ^a^	1.32 ± 0.02 ^a^	1.92 ± 0.07 ^b^
Pro	NEAA	2.79 ± 0.07 ^b^	0.14 ± 0.04 ^a^	0.24 ± 0.03 ^a^	0.06 ± 0.02 ^a^
Asp	NEAA	28.10 ± 0.19 ^a^	51.06 ± 0.16 ^c^	31.05 ± 0.17 ^b^	63.57 ± 0.05 ^d^
Phe	EAA	0.82 ± 0.03 ^a^	0.46 ± 0.09 ^a^	0.67 ± 0.05 ^a^	0.66 ± 0.02 ^a^
Lys	EAA	1.82 ± 0.06 ^c^	3.02 ± 0.09 ^d^	1.56 ± 0.03 ^b^	0.25 ± 0.02 ^a^
Glu	NEAA	1.09 ± 0.05 ^a^	3.17 ± 0.05 ^c^	1.16 ± 0.01 ^a^	2.56 ± 0.01 ^b^
∑ TAA		56.82 ± 0.89 ^a^	87.37 ± 1.15 ^c^	60.58 ± 1.01 ^b^	94.38 ± 0.73 ^d^
∑ EAA		12.25 ± 0.43 ^d^	9.95 ± 0.66 ^b^	10.86 ± 0.32 ^c^	7.37 ± 0.35 ^a^
∑ EAA/TAA		0.22 ^b^	0.11 ^a^	0.18 ^b^	0.08 ^a^

JS: Jonathan skin; JP: Jonathan pomace; GS: Golden Delicious skin; GP: Golden Delicious pomace; different superscript letters in a row indicate significant difference between samples (*p* < 0.05).

**Table 3:** Apple by-products volatile aroma compounds.

Volatile Compounds	JS	JP	GS	GP	Odor Perception
Alcohols					
1-Pentanol	n.d.	0.12 ± 0.02 ^a^	n.d	0.38 ± 0.03 ^ab^	Pungent, fermented, bready, fusel, wine, solvent
2-methyl-1-butanol	0.75 ±0.03 ^a^	0.80 ± 0.03 ^a^	1.12 ±0.02 ^b^	1.26 ± 0.02 ^b^	Acidic, sharp, spicy, fusel, wine
1-butanol	2.93 ± 0.03 ^c^	1.25 ± 0.05 ^b^	0.27 ± 0.02 ^a^	n.d.	Sweet, balsamic, oily, whiskey
1-octanol	0.29 ± 0.02 ^ab^	0.79 ± 0.02 ^c^	0.13 ± 0.01 ^a^	0.45 ±0.01 ^b^	Herbal, waxy, fruity nuance
(Z)-hexen-3-ol	0.22 ± 0.02 ^ab^	n.d.	0.13 ± 0.01 ^a^	n.d.	Fresh, green, raw fruity with a pungent depth
1-hexanol	2.18 ± 0.11 ^a^	3.73 ± 0.02 ^b^	2.50 ± 0.02 ^a^	4.11 ± 0.03 ^c^	Green, sweet, herbaceous, fermented note, fruity, apple skin, and oily
**Total**	6.37 ± 0.04 ^b^	6.69 ± 0.04 ^bc^	4.15 ± 0.03 ^a^	6.20 ± 0.01 ^b^	
Esters					
Ethyl hexanoate	5.29 ± 0.13 ^d^	3.51 ± 0.02 ^c^	0.11 ± 0.02 ^a^	1.23 ± 0.03 ^b^	Fruity, apple peel fruits, pineapple, green banana nuance, waxy, fatty
Ethyl butanoate	0.57 ± 0.03 ^a^	5.19 ± 0.04 ^c^	n.d.	0.94 ± 0.03 ^ab^	Fruity, pineapple, apple, cognac
Ethyl 2-methylbutanoate	n.d.	0.12 ± 0.02 ^a^	1.8 ± 0.02 ^c^	1.14 ± 0.03 ^b^	Sharp sweet, fruity, green, apple peel, pineapple skin
Butyl acetate	19.76 ± 0.05 ^d^	3.18 ±0.05 ^a^	16.20 ± 0.03 ^c^	3.83 ± 0.04 ^ab^	Sweet, ripe banana, ethereal
2-methylbutanoate	0.21 ± 0.03 ^a^	0.32 ± 0.02 ^a^	n.d.	0.79 ± 0.03 ^b^	Fruity, apple, fresh pear, and tropical nuance
2-methylbutyl acetate	9.23 ± 0.04 ^b^	17.57 ± 0.03 ^d^	4.93 ± 0.05 ^a^	12.04 ± 0.06 ^c^	Sweet, fruity, ripe banana, pear, apple
Hexyl acetate	7.18 ± 0.21 ^c^	4.26 ± 0.07 ^a^	6.30 ± 0.04 ^b^	4.71 ± 0.04 ^a^	Fresh, fruity, apple, pear, and banana note
Butyl-butyrate	n.d.	0.80 ± 0.02 ^ab^	n.d.	0.49 ± 0.03 ^a^	Sweet, fresh, fruity, slightly fatty
Butyl 2-methylbutanoate	2.11 ± 0.03 ^d^	1.29 ± 0.03 ^b^	1.70 ± 0.02 ^c^	0.85 ± 0.02 ^a^	Fruity, apple, tropical, cocoa
Butyl hexanoate	n.d.	0.61 ± 0.03 ^ab^	n.d.	0.14 ± 0.02 ^a^	Fruity, pineapple, waxy, green, juicy
2-methylbutyl 2-methylbutanoate	4.11 ± 0.05 ^d^	0.83 ± 0.03 ^a^	2.54 ± 0.04 ^c^	1.36 ± 0.05 ^b^	Fruits, apple, with green, waxy, and woody nuances
Hexyl butanoate	0.81 ± 0.02 ^c^	0.34 ± 0.02 ^ab^	0.11 ± 0.05 ^a^	n.d.	Green, sweet, fruity, apple waxy, wine
Hexyl 2-methylbutanoate	3.59 ± 0.06 ^b^	3.12 ± 0.22 ^a^	11.12 ± 0.11 ^c^	16.30 ± 0.21 ^d^	Green, waxy, fruity, apple, banana, and woody with a tropical, spicy nuance
Hexyl hexanoate	0.43 ± 0.11 ^a^	0.41 ± 0.03 ^a^	n.d.	3.98 ± 0.05 ^b^	Fruity, wine, orange peel, apple, cucumber
**Total**	53.29 ± 0.76 ^d^	41.55 ± 0.63 ^a^	44.81 ± 0.38 ^b^	47.80 ± 0.64 ^c^	
Aldehydes					
Hexanal	13.17 ± 0.03 ^a^	17.58 ± 0.05 ^c^	20.68 ± 0.23 ^d^	14.11 ± 0.06 ^b^	Intense green, fruity, aldehydic odor, green apple
Furfural	0.71 ± 0.05 ^a^	1.25 ± 0.05 ^b^	1.88 ± 0.02 ^c^	3.32 ± 0.04 ^d^	Caramel, bitter almond, nutty, baked bread
2-hexenal	0.96 ± 0.03 ^a^	2.79 ± 0.02 ^b^	4.65 ± 0.02 ^c^	5.72 ± 0.03 ^d^	Fruity, green leaf, apple
Heptanal	0.80 ± 0.03 ^a^	1.95 ± 0.04 ^c^	1.92 ± 0.02 ^c^	1.15 ± 0.04 ^b^	Green, oily, citrus
2-heptenal	2.85 ± 0.02 ^a^	3.13 ± 0.02 ^b^	4.27 ± 0.03 ^c^	2.79 ± 0.03 ^a^	Intense green, sweet, oily, apple skin nuances, fruity overtones
Benzaldehyde	4.21 ± 0.02 ^c^	6.21 ± 0.03 ^d^	0.19 ± 0.04 ^a^	1.55 ± 0.05 ^b^	Almond, fruity, powdery, nutty
Octanal	1.91 ± 0.02 ^a^	4.17 ± 0.07 ^c^	1.81 ± 0.03 ^a^	3.38 ± 0.04 ^b^	Green, fat, citrus peel
E-2-octenal	3.77 ± 0.03 ^c^	2.70 ± 0.02 ^b^	2.54 ± 0.06 ^b^	1.61 ± 0.04 ^a^	Honey, green, fatty, walnut
Nonanal	0.35 ± 0.04 ^a^	2.18 ± 0.03 ^c^	0.85 ± 0.01 ^b^	4.24 ± 0.05 ^d^	Green, floral, sweet orange, rose, waxy
Decanal	1.02 ± 0.02 ^b^	2.03 ± 0.05 ^c^	0.74 ± 0.03 ^ab^	0.54 ± 0.02 ^a^	Waxy, fatty, citrus peel, green melon nuance
**Total**	29.75 ± 0.27 ^a^	43.99 ± 0.38 ^d^	39.53 ± 0.49 ^c^	38.41 ± 0.40 ^b^	
Ketones					
Acetophenone	2.88 ± 0.03 ^d^	2.08 ± 0.02 ^c^	1.12 ± 0.05 ^b^	0.84 ± 0.03 ^a^	Floral, almond, nutty, must, spicy
1-octen-3-one	n.d.	n.d	1.34 ± 0.02 ^a^	1.93 ± 0.03 ^b^	Mushroom, herbal, earthy
6-methyl-5-hepten-2-one	2.52 ± 0.04 ^ab^	2.06 ± 0.03 ^a^	3.26± 0.03 ^c^	2.13 ± 0.05 ^a^	Citrus, green, musty, lemongrass, apple, bittersweet taste
**Total**	5.40 ± 0.06 ^c^	4.14 ± 0.05 ^a^	5.72 ± 0.10 ^d^	4.90 ± 0.11 ^ab^	
Terpenes and terpenoids					
Camphene	0.31 ± 0.02 ^ab^	0.21 ± 0.01 ^a^	1.10 ± 0.03 ^c^	0.17 ± 0.02 ^a^	Camphoraceous, green spicy nuances
Sabinene	0.15 ± 0.01 ^ab^	0.07 ± 0.02 ^a^	0.11 ± 0.02 ^ab^	n.d.	Woody, citrus, oily, fruity, pine, spice nuance
*ß*-pinene	n.d.	n.d.	0.40 ± 0.02 ^a^	0.36 ± 0.02 ^a^	Woody, pine, resinous, camphoreous balsamic, spicy
*ß* -myrcene	n.d.	n.d.	0.35 ± 0.03 ^a^	0.20 ± 0.01 ^a^	Herbaceous, woody, spice, balsamic
3-carene	n.d.	n.d.	0.50 ± 0.02	n.d.	Harsh, terpene-like, coniferous
1,3,8-p-menthatriene	0.47 ± 0.01 ^b^	0.20 ± 0.02 ^a^	1.09 ± 0.03 ^c^	0.18 ± 0.03 ^a^	Camphor, herbal, turpentine, woody
*p*-cymene	0.49 ± 0.02 ^ab^	0.35 ± 0.02 ^ab^	0.23 ± 0.02 ^a^	n.d.	Solvent, citrus, woody, spicy
D-limonene	1.50 ± 0.03 ^b^	1.27 ± 0.04 ^b^	0.30 ± 0.02 ^a^	1.16 ± 0.05 ^b^	Citrus, fresh, sweet
γ-terpinene	n.d.	n.d	0.24 ± 0.02	n.d	Herbal, citrus, lemon, spicy
Terpinolene	0.05 ± 0.02 ^a^	n.d	0.07 ± 0.01 ^a^	n.d	Sweet, fresh, piney, old lemon peel nuance
*ß*- linalool	0.10 ± 0.01 ^a^	0.26 ± 0.02 ^ab^	n.d	n.d	Fresh, floral-woody, sweet, citrus
α-farnesene	1.54 ± 0.03 ^d^	0.40 ± 0.03 ^ab^	0.75 ± 0.04 ^c^	0.20 ± 0.01 ^a^	Wood, sweet, floral
**Total**	4.61 ± 1.04 ^c^	2.76 ± 1.05 ^ab^	5.14 ± 1.15 ^d^	2.27 ± 0.14 ^a^	
Acids					
Benzoic acid	0.06 ± 0.02 ^a^	0.45 ± 0.03 ^b^	0.36 ± 0.02 ^b^	0.12 ± 0.04 ^ab^	Fade balsamic
2-methylbutanoic acid	n.d.	0.11 ± 0.05 ^a^	0.29 ± 0.03 ^ab^	0.30 ± 0.02 ^ab^	Acidic, fruity, fatty, cheesy with fermented nuance
**Total**	0.06 ± 0.02 ^a^	0.56 ± 0.03 ^ab^	0.56 ± 0.02 ^ab^	0.42 ± 0.02 ^ab^	
Others					
2-pentyl furan	0.52 ± 0.02 ^a^	0.31 ± 0.01 ^a^	n.d.	n.d.	Green, earthy, beans, musty, cooked, caramel like
**Total**	0.52 ± 0.02 ^a^	0.31 ± 0.01 ^a^	n.d.	n.d.	

JS: Jonathan skin; JP: Jonathan pomace; GS: Golden Delicious skin; GP: Golden Delicious pomace; different superscript letters in a row indicate significant difference between samples (*p* < 0.05).

**Table 4:** Apple biscuits and by-products color characteristics.

Color Parameters			
Samples	L*	a*	b*
BCS	68.50 ± 0.11 ^e^	5.70 ± 0.09 ^a^	30.48 ± 0.13 ^b^
BJS	51.25 ± 0.55 ^a^	12.32 ± 0.72 ^d^	27.64 ± 0.55 ^a^
BJP	59.31 ± 0.66 ^c^	10.79 ± 0.55 ^c^	31.89 ± 0.22 ^c^
BGS	58.44 ± 0.28 ^b^	10.86 ± 0.19 ^c^	39.05 ± 0.55 ^e^
BGP	64.47 ± 0.33 ^d^	9.00 ± 0.07 ^b^	36.96 ± 0.91 ^d^
JS	59.93 ± 0.05 ^A^	12.46 ± 0.04 ^D^	15.22 ± 0.08 ^A^
JP	71.8 ± 0.32 ^B^	9.22 ± 0.06 ^C^	19.05 ± 0.12 ^B^
GS	78.52 ± 0.22 ^C^	0.46 ± 0.03 ^A^	29.50 ± 0.33 ^D^
GP	80.96 ± 0.17 ^D^	2.82 ± 0.05 ^B^	27.64 ± 0.55 ^C^

BCS: biscuits control sample; BJS: biscuits with JS; BJP: biscuits with JP; BGS: biscuits with GS; BGP: biscuits with GP; JS: Jonathan skin; JP: Jonathan pomace; GS: Golden skin; GP: Golden pomace; L* (luminosity), a* (red/green coordinate), b* (yellow/blue coordinate) color; different small superscript letters in a column indicate significant difference between final baked goods (*p* < 0.05), meantime, different big superscript letters in a column indicate significant difference between apple by-products.

**Table 5:** Biscuit’s recipes and technological parameters.

Ingredients (g)	Biscuits Samples				
	BCS	BJS	BJP	BGS	BGP
Wheat flour (WF)	100	-	-	-	-
JS	75	25	-	-	-
JP	75	-	25	-	-
GS	75	-	-	25	-
GP	75	-	-	-	25
Vegetable fat	40	40	40	40	40
Powdered milk	20	20	20	20	20
Sugar	30	30	30	30	30
Baking powder	2.5	2.5	2.5	2.5	2.5
Water	25	25	25	25	25
**Technological Parameters**					
Mixing time (minutes)	7	7	7	7	7
Dough temperature (°C)	20	20.5	20.3	21.0	20.5
Resting time (minutes)	45	45	45	45	45
Temperature (°C)	4–6	4–6	4–6	4–6	4–6
Baking time (minutes)	15	15	15	15	15
Temperature (°C)	180	180	180	180	180

BCS: control sample; BJS: biscuits with JS; BJP: biscuits with JP; BGS: biscuits with GS; BGP: biscuits with GP; JS: Jonathan skin; JP: Jonathan pomace; GS: Golden skin; GP: Golden pomace.

## Data Availability

Not applicable.

## Supplementary Materials

The following supporting information can be downloaded at: https://www.mdpi.com/article/10.3390/molecules27061987/s1, Table S1. Biscuits volatile aroma compounds. Table S2. Used reagents list.

## Sample Availability

Samples of the compounds are not available from the authors.
