# Supplementary material for: Analysis of Fatty Acids, Amino Acids and Volatile Profile of Apple By-Products by Gas Chromatography-Mass Spectrometry
Source: Molecules. 2022 Mar 19;27(6):1987. doi: 10.3390/molecules27061987 (PMC8955822; doi:10.3390/molecules27061987)
Supplement: Supplementary file 1 [file molecules-27-01987-s001.zip › molecules-1638168-supplementary.pdf]

Table S1. Biscuits volatile aroma compounds

Volatile compounds	BCS	BJS	BJP	BGS	BGP	Odour perception
**Alcohols**						
1-hexanol	n.d.	0.80 ± 0.02[a]	1.30 ± 0.02[b]	2.10 ± 0.02[c]	2.70 ± 0.03[d]	Green, sweet, herbaceous, fermented note, fruity, apple-skin and oily
Phenol	1.03 ± 0.02	n.d.	n.d.	n.d.	n.d.	Phenol
**Total**	1.03 ± 0.02[b]	0.80 ± 002[a]	1.30 ± 0.02[bc]	2.10 ± 0.02[d]	2.70 ± 0.03[e]	
**Esters**						
Butyl acetate	n.d.	12.66 ± 0.04[d]	12.01 ± 0.03[c]	11.09 ±0.05[b]	10.23 ± 0.03a	Sweet, ripe banana, ethereal
Hexyl acetate	5.23 ± 0.04[a]	14.80 ± 0.04[e]	10.89 ± 0.07[b]	13.90 ± 0.05[d]	11.02 ± 0.07[c]	Fresh, fruity, apple, pear and banana note
2-methylbutyl acetate	0.34 ± 0.03[a]	3.44 ± 0.22[b]	3.67 ± 0.21[b]	4.09 ± 0.04[c]	5.03 ± 0.03[d]	Sweet, fruity, ripe banana, pear, apple
**Total**	5.57 ± 0.07	30.90 ± 0.30	26.57 ± 0.31	29.08 ± 0.15	26.28 ± 0.13	
**Aldehydes**						
Benzaldehyde	2.90 ± 0.05[a]	9.99 ± 0.11[e]	8.05 ± 0.23[d]	5.03 ± 0.11[c]	4.08 ± 0.22[b]	Almond, fruity, powdery, nutty
Hexanal	53.21 ± 0.20[e]	7.05 ± 0.10[a]	11.55 ± 0.21[b]	12.03 ± 0.13[c]	13.53 ± 0.15[d]	Intense green, aldehydic odor, off flavor
Nonanal	7.12 ± 0.03[c]	3.04 ± 0.02[a]	3.55 ± 0.04[ab]	7.25 ± 0.05[c]	8.01 ± 0.06[d]	Green, floral, sweet orange, rose, waxy
Phenylacetaldehyde	n.d.	6.01 ± 0.05[a]	6.11 ± 0.09[a]	6.42 ± 0.04[a]	6.75 ± 0.05[ab]	Fatty, fruity, cake crust, bready
Methional	n.d.	1.55 ± 0.03[a]	2.01 ± 0.21[b]	3.55 ± 0.07[c]	5.55 ± 0.08[d]	Potato, damp
3-methyl-butanal	1.67 ± 0.05[ab]	4.22 ± 0.03[e]	3.77 ± 0.22[d]	2.01 ± 0.11[c]	1.09 ± 0.13[a]	Dried fruits, nutty, cocoa, chocolate, fatty
2-methyl-butanal	1.99 ± 0.02[a]	4.33 ± 0.03[d]	3.87 ± 0.02[c]	5.03 ± 0.03[d]	2.66 ± 0.05[b]	Malty, cacao, chocolate, coffee, caramellike, nutty, rummy, malty
2-methyl-propanal	0.76 ± 0.03[a]	1.65 ± 0.02[b]	2.55 ± 0.04c	1.89 ± 0.05[b]	3.67± 0.03[d]	Wine, solvent, malty, fruity
**Total**	67.57 ± 0.38[e]	37.84 ± 0.39[a]	41.46 ± 1.02[b]	43.21 ± 0.59[c]	45.34 ± 0.77[d]	
**Ketones**						
2-heptanone	0.88 ± 0.13[a]	13.99 ± 0.22[e]	13.27 ± 0.37[d]	11.77 ± 0.03[c]	10.57 ± 0.33[b]	Cheese, fruity, ketonic, green banana, with a creamy nuance
Acetophenone	4.30 ± 0.03[a]	8.75 ± 0.05[e]	7.77 ± 0.06[d]	6.11 ± 0.03[c]	5.21 ± 0.02[b]	Floral, almond, nutty, must, spicy
**Total**	5.18 ± 0.16[a]	22.74 ± 0.27[e]	21.04 ± 0.43[d]	17.88 ± 0.06[c]	15.78 ± 0.35[b]	
**Terpens and terpenoids**						

ß -myrcene	0.79 ± 0.02^{a}	n.d.	1.39 ± 0.03^{b}	n.d.	3.78 ± 0.05^{c}	Herbaceous, woody, spice, balsamic
D-limonene	0.69 ± 0.03^{b}	2.99 ± 0.04^{d}	2.55 ± 0.02^{c}	0.30 ± 0.01^{a}	0.20 ± 0.02^{a}	Citrus, fresh, sweet
**Total**	1.48 ± 0.05^{b}	2.99 ± 0.04^{c}	3.94 ± 0.05^{d}	0.30 ± 0.01^{a}	3.96 ± 0.07^{d}	
**Acids**						
Benzoic acid	15.03 ± 0.22^{e}	0.30 ± 0.02^{a}	1.64 ±0.03^{b}	3.20 ± 0.04^{c}	3.67 ± 0.03^{d}	Fade balsamic
**Total**	15.03 ± 0.22^{e}	0.30 ± 0.02^{a}	1.64 ±0.03^{b}	3.20 ± 0.04^{c}	3.67 ± 0.03^{d}	
**Others**						
Ethyl 2,4-dioxohexanoate	2.10 ± 0.05^{a}	4.50 ±0.03	4.05 ± 0.06^{b}	4.23 ± 0.07^{b}	2.25 ± 0.05^{a}	Apple peel, fruit
Dimethyl disulfide	1.96 ± 0.07	n.d.	n.d.	n.d.	n.d.	Vegetal, sulfurous, cabbage, malt
**Total**	4.06 ± 0.12^{b}	4.50 ±0.03^{b}	4.05 ± 0.06^{b}	4.23 ± 0.07^{b}	2.25 ± 0.05^{a}	

Table S2. Used reagents list

Used reagent	Determination	Company, city, country)
Methanol	Fatty acids	Sigma Aldrich (Steinheim, Germany)
Chloroform	Fatty acids	Sigma Aldrich (Steinheim, Germany)
Potassium chloride	Fatty acids	Sigma Aldrich (Steinheim, Germany)
Sodium sulphate	Fatty acids	Sigma Aldrich (Steinheim, Germany)
Trichloroacetic acid	Amino-acids	Sigma Aldrich (Steinheim, Germany)
15N-glycine 99 atom %	Amino-acids	Sigma Aldrich (Steinheim, Germany)
